# Clinical Reactivations of Herpes Simplex Virus Type 2 Infection and Human Immunodeficiency Virus Disease Progression Markers

**DOI:** 10.1371/journal.pone.0009973

**Published:** 2010-04-01

**Authors:** Bulbulgul Aumakhan, Charlotte A. Gaydos, Thomas C. Quinn, Chris Beyrer, Lorie Benning, Howard Minkoff, Daniel J. Merenstein, Mardge Cohen, Ruth Greenblatt, Marek Nowicki, Kathryn Anastos, Stephen J. Gange

**Affiliations:** 1 Johns Hopkins Bloomberg School of Public Health, Baltimore, Maryland, United States of America; 2 Johns Hopkins University School of Medicine, Baltimore, Maryland, United States of America; 3 Laboratory of Immunoregulation, Division of Intramural Research, National Institute of Allergy and Infectious Diseases, National Institutes of Health, Bethesda, Maryland, United States of America; 4 Maimonides Medical Center and SUNY Downstate, Brooklyn, New York, United States of America; 5 Georgetown University Medical Center, Washington, D. C., United States of America; 6 Cook County Medical Center, Chicago, Illinois, United States of America; 7 Schools of Pharmacy and Medicine, University of California San Francisco, San Francisco, California, United States of America; 8 University of Southern California, Los Angeles, California, United States of America; 9 Albert Einstein College of Medicine, Bronx, New York, United States of America; University of Cape Town, South Africa

## Abstract

**Background:**

The natural history of HSV-2 infection and role of HSV-2 reactivations in HIV disease progression are unclear.

**Methods:**

Clinical symptoms of active HSV-2 infection were used to classify 1,938 HIV/HSV-2 co-infected participants of the Women's Interagency HIV Study (WIHS) into groups of varying degree of HSV-2 clinical activity. Differences in plasma HIV RNA and CD4+ T cell counts between groups were explored longitudinally across three study visits and cross-sectionally at the last study visit.

**Results:**

A dose dependent association between markers of HIV disease progression and degree of HSV-2 clinical activity was observed. In multivariate analyses after adjusting for baseline CD4+ T cell levels, active HSV-2 infection with frequent symptomatic reactivations was associated with 21% to 32% increase in the probability of detectable plasma HIV RNA (trend p = 0.004), an average of 0.27 to 0.29 log10 copies/ml higher plasma HIV RNA on a continuous scale (trend p<0.001) and 51 to 101 reduced CD4+ T cells/mm^3^ over time compared to asymptomatic HSV-2 infection (trend p<0.001).

**Conclusions:**

HIV induced CD4+ T cell loss was associated with frequent symptomatic HSV-2 reactivations. However, effect of HSV-2 reactivations on HIV disease progression markers in this population was modest and appears to be dependent on the frequency and severity of reactivations. Further studies will be necessary to determine whether HSV-2 reactivations contribute to acceleration of HIV disease progression.

## Introduction

Individuals with human immunodeficiency virus (HIV) infection are often found to be co-infected with herpes simplex virus type 2 (HSV-2) – the causative agent of genital herpes (GH).

Genital herpes has a widely variable clinical course. Some individuals manifest severe forms of disease with the frequent development of painful and typical mucocutaneous lesions while others experience mild or atypical forms with manifestations that can easily go unnoticed [1]. In rare situations, the herpes virus can disseminate systemically and cause devastating complications in many internal organs [2]–[4]. The effect of this clinical heterogeneity on the course of HIV is not clear.

The nature and precise mechanisms of interaction between these two sexually transmitted pathogens are still incompletely understood with studies often producing conflicting results leading to ongoing debate on whether HSV-2 is indeed a significant risk factor for HIV acquisition, transmission or disease progression. Some experts argue that there is sufficient evidence of viral synergy between these two sexually transmitted pathogens to call for immediate actions to control HSV-2 [5]. Others argue that recent trials demonstrating no effect of HSV suppressive therapy on HIV acquisition and transmission [6]–[8] suggest that HSV-2 based interventions will have little to no effect on HIV epidemic [9].

We attempted to investigate this interaction within Women's Interagency HIV Study (WIHS), the nation's largest cohort study of HIV natural history by evaluating the association of the type (symptomatic vs. asymptomatic) and severity (frequency of symptomatic recurrences) of HSV-2 infection with HIV disease progression markers (plasma HIV RNA and CD4+ T cell count) among highly active antiretroviral therapy (HAART) naïve HIV/HSV-2 co-infected participants of WIHS.

## Methods

### Ethics Statement

This study was conducted under IRB approval of WIHS Data Management and Analysis Center (Principal Investigator – Stephen Gange). IRB number: H.34.97.05.19.A2. Written informed consent was obtained from all participants.

### Study population

The study population consisted of 2,056 HIV infected participants recruited into WIHS between 1994 and 1995. WIHS is an ongoing multicenter cohort study of HIV natural history in women across six sites in the U.S. (Los Angeles, CA; Washington, DC; the San Francisco Bay area, CA; New York City/Bronx, NY; Brooklyn, NY; and Chicago, IL). Complete physical and gynecological examination with collection of biologic specimens was done on women semiannually since 1994. Gynecological examination included assessment for genital tract infections, including ulcers and intraepithelial dysplasia as previously described [10]. Detailed review of the WIHS study population and methods have been previously published [11].

### Study follow up

Study follow up was restricted to the first three WIHS visits (1.5 years of follow up). Less than 3% of participants were treated with HAART during that time period. To avoid possible selection and survival biases participants with only one (n = 221) or two (n = 237) study visits as well as those on HAART (n = 40) were excluded.

### Exposure assessment

#### 1) Laboratory assessment of HSV serostatus

Serologic reactivity to HSV-1 and HSV-2 was determined by a commercially available type-specific glycoprotein G-based enzyme immunoassay (gG-EIA, Gull Laboratories, Salt Lake City, Utah, USA) at enrollment as previously described [12], [13]. Negative and equivocal results were confirmed by Western Blot as previously described [13]–[15]. The sensitivity of the gG-EIA for HSV-1 is 95% and the specificity is 96%. For HSV-2, the sensitivity is 98% and the specificity is 97% [12], [13].

#### 2) Clinical assessment of HSV-2 infection and classification of study groups

Clinicians' assessment of genital herpes was based on visualization of ulcers and/or vesicles in the genital area during pelvic exam. Clinicians were either women's health nurse practitioners or physician gynecologists. All have been trained to complete the WIHS protocol. An interval history of genital sores was collected by self-report at each study visit. Participants were classified into groups based on the type of HSV-2 infection (symptomatic or asymptomatic) and frequency of visits with genital lesions observed over the duration of the initial three visits. This classification was based on the assumption that the likelihood of having active and more severe HSV-2 infection will be highest in clinically symptomatic women presenting with herpetic lesions at least once during the specified study period as opposed to women who remain clinically asymptomatic during the same time period. This assumption also stems from the results of preliminary work in which the presence and quantity of HSV-2 DNA in lower genital tract was highest in symptomatic women presenting with typical herpetic lesions (∼30%) compared to women clinically asymptomatic over extended periods of time or women with other lesions (<10%) when cervicovaginal lavage samples from a subset of WIHS women (n∼400) were tested by polymerase chain reaction (Aumakhan, unpublished thesis). Based on this assumption and data, a participant who was HSV-2 seropositive but negative for potential indicators of active HSV-2 infection, such as presence of any genital lesions and negative for a history of genital sores over the duration of this study was considered as having asymptomatic HSV-2 infection and considered as having symptomatic HSV-2 infection if she had at least one visit (out of three) at which she presented with genital lesions identified by clinicians as herpetic and/or had a positive history of GH sores in between the visits. The degree of HSV-2 clinical activity among symptomatic women was then graded from: a) mild symptomatic (only self report positive but no lesions at any study visit), b) mild to moderate (lesions at only one of the study visits), c) moderate (lesions at two visits), and d) severe symptomatic (lesions at all three study visits). Women with lesions not identified as herpetic and negative for a history of genital sores were considered as having symptoms not highly suggestive of active HSV-2 infection and thus, excluded.

### Outcome assessment

HIV RNA plasma load was quantified by an isothermal nucleic acid sequence-based amplification assay (NASBA/Nuclisens, Merieux, Durham, North Carolina, USA), which had a lower limit of quantification (LOQ) of 4,000 copies/ml. CD4+ T cells were quantified using standard flow cytometry methods.

### Statistical analysis

Plasma HIV RNA load was stratified into two categories with the cutpoint at the LOQ: ≤4,000 and >4,000. HIV RNA values below the LOQ were summarized using proportions, and values above the LOQ were log_10_ transformed for comparison purposes. Univariate analyses were carried out comparing selected characteristics between defined study groups. Proportions were compared using the chi square test. Continuous variables were summarized with medians and compared using the Wilcoxon rank-sum (Mann-Whitney) test. The Generalized Estimating Equations (GEE) method with robust variance estimation was used to explore the mean differences in plasma HIV RNA and CD4+ T cell counts between the study groups. To explore the cumulative association of repeated HSV-2 episodes on HIV markers, linear regression for continuous and Poisson extensions of log binomial models [16] for dichotomous outcomes were implemented at the third or last follow up visit. Multivariate models adjusted for selected demographic and risk variables known to be associated with HIV risk and disease progression as well as variables that were statistically different in crude comparisons and included age, race, injection and noninjection drug use, number of lifetime sexual partners, use of herpes medications and antiretroviral therapy, as well as baseline CD4+ T cell count. The asymptomatic group was the reference group in all models. Analyses were carried out with STATA, version 10 (Stata Corporation, College Station, Texas, USA). Statistical significance was defined as a p-value of <0.05.

## Results

### a) Study groups

**Figure 1:**
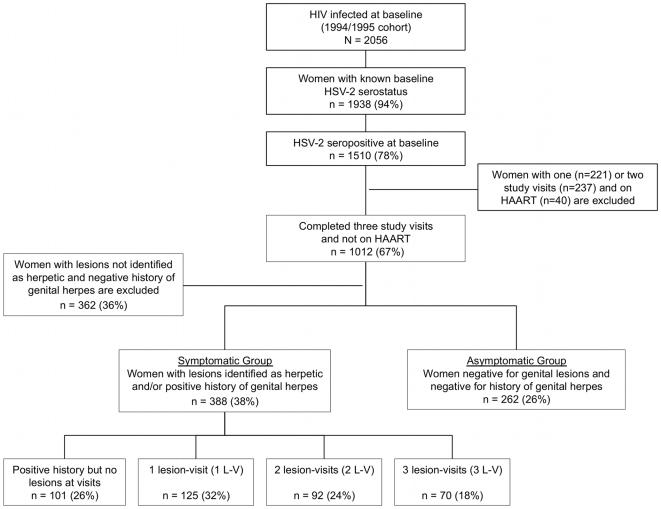
Flowchart of study participants and description of exposure groups.

Ninety four percent of the source cohort (n = 1,938) had known baseline HSV-2 serostatus and of those 78% (n = 1,510) were HSV-2 seropositive (Figure 1). After excluding women with incomplete follow up as well as those on HAART there were 1,012 women eligible for the study. Of 1,012 women, 262 (n = 26%) were classified as asymptomatic for HSV-2 infection and 388 (n = 38%) as symptomatic according to the definitions described earlier. Women with lesions not identified by clinicians as herpetic as well as negative for an interval history of genital sores (n = 362, 36%) were excluded. By degree of HSV-2 clinical activity, of 388 symptomatic women 101 (26%) had positive history of GH but no lesions identified at any of the three study visits, 125 (32%) presented with genital lesions once (1 lesion-visit or 1 L-V); 92 (24%) - twice (2 lesion-visits or 2 L-V); and 70 (18%) had lesions observed at all three visits (3 lesion-visits or 3 L-V).

### b) Comparison of demographic, risk and clinical characteristics by study groups

Table 1 displays distribution of selected baseline characteristics by main study groups: symptomatic vs. asymptomatic. Overall, symptomatic women were younger than asymptomatic women (median age 36 years vs. 38 years, p = 0.001). White women tended to be symptomatic as opposed to their African American counterparts (71% vs. 57%, p = 0.026). In addition, symptomatic women were more likely to be on combination antiretroviral therapy (35% vs. 26%, p = 0.013). However, the biggest difference was observed in the use of herpes treatment: 19% of symptomatic women vs. 3% of asymptomatic women reported use of herpes medications at baseline visit (p<0.0001). There were no statistically significant differences observed in HIV risk exposure, number of sexual partners, or proportion of HSV-1 seropositivity between the groups. Demographic, risk and clinical characteristics were not significantly different when explored by subgroups of HSV-2 clinical activity or the frequency of lesion-visits within the symptomatic cohort.

**Table 1:** Comparison of demographic, risk and clinical characteristics by study groups.

Characteristic/Study Group		Asymptomatic (n = 262)	Symptomatic (n = 388)	*P*
Median age at baseline (IQR), years		38 (33–43)	36 (31–40)	0.001
Median age at 1^st^ sex (IQR), years		15 (13–17)	15 (13–17)	0.459
Race/Ethnicity, n (%)	African-American	175 (67%)	228 (59%)	0.026
	Latina/Hispanic	53 (20%)	91 (23%)	
	White	24 (9%)	61 (16%)	
	Other	10 (4%)	8 (2%)	
HIV risk exposure category, n (%)	Intravenous drug use	98 (38%)	116 (30%)	0.183
	Heterosexual risk	96 (37%)	166 (43%)	
	Transfusion risk	8 (3%)	17 (4%)	
	Not known	60 (23%)	89 (23%)	
Number of lifetime sexual partners, n (%)	0–1	8 (3%)	11 (3%)	0.428
	2–4	44 (17%)	47 (12%)	
	5–9	42 (16%)	69 (18%)	
	10–49	85 (33%)	121 (31%)	
	≥50	81 (31%)	138 (36%)	
	Not known	2 (<1%)	2 (<1%)	
HSV-1 seropositive, n (%)		204 (78%)	277 (71%)	0.065
Use of herpes treatment at baseline, n (%)		8 (3%)	74 (19%)	<0.001
Use of antiretroviral therapy at baseline, n (%)	None	113 (43%)	128 (33%)	0.013
	Mono	82 (31%)	125 (32%)	
	Combination	67 (26%)	135 (35%)	

Note: IQR, interquartile range; *P*, p-value.

### c) Comparison of HIV disease progression markers by study groups

Table 2 presents values of HIV markers by the main study groups as well as by subgroups of HSV-2 clinical activity. A gradient trend was observed such that the values of HIV markers became progressively worse as the level of HSV-2 clinical activity progressively increased from mild symptomatic to severe symptomatic with frequent reactivations. Specifically, symptomatic women with outbreaks of genital lesions at all three visits had more than 3-fold unfavorable differences in outcome HIV markers compared to asymptomatic women who had none (p<0.001). Interestingly, among these symptomatic women only 5 had lesions identified as herpetic at all three visits but they had the highest viral load values with median plasma HIV RNA of 490,000 copies/ml (interquartile range [IQR]: 205,000–3,865,000) and lowest CD4 T cell counts with median CD4+ T cell number of only 14 (IQR: 4–94).

**Table 2:** Comparison of baseline HIV disease markers by study groups.

Characteristic/Study Group	Asymptomatic (N = 262)	Symptomatic (N = 388)				*P* *
		SR GH^†^ (N = 101)	1 L–V^‡^ (N = 125)	2 L–V (N = 92)	3 L–V (N = 70)	
Number/Total (%) with HIV pVL ≤4,000 copies/ml	107/258 (41%)	36/99 (36%)	34/125 (27%)	22/88 (25%)	9/69 (13%)	<0.001
Median HIV pVL (IQR), copies/ml**	25,000 (12,000–100,000)	42,000 (13,000–1000,000)	46,000 (19,000–140,000)	76,500 (23,000–180,000)	92,000 (20,500–245,000)	<0.001
Median CD4+ T cell count (IQR), cells/mm^3^	418 (285–593)	391 (270–567)	347 (179–542)	282 (147–430)	138 (19–365)	<0.001

Note: IQR, interquartile range; SR GH, self reported genital herpes; L-V, lesion-visit; ^†^positive self report of genital herpes only; ^‡^number of visits with lesions.

* *P* - *p-value* for overall comparison of symptomatic group vs. asymptomatic group.

** among women with HIV pVL >4,000 copies/ml.

### d) Association between frequency of lesion-visits and the probability of detectable plasma HIV RNA

Crude GEE analyses demonstrated a 21%, 25% and 50% increased probability of detectable plasma HIV RNA in women with lesions at one, two and three visits respectively (Table 3). Corresponding adjusted estimates were 11%, 13% and 21% (Table 4, trend p-value  = 0.005). Similarly, an 8% to a 32% increase was observed when Poisson extension of log binomial regression was carried out to estimate the cumulative effect of repeated lesion-visits on the probability of detectable plasma HIV RNA at the last or 3^rd^ study visit (Table 5, trend p-value  = 0.004).

**Table 3:** Crude prevalence risk ratios (PRs) of detectable plasma HIV RNA and mean differences in plasma HIV RNA and CD4+ T cell counts (from the GEE analysis with robust variance estimation) by degree of HSV-2 clinical activity.

		Detectable plasma HIV RNA (>4,000 copies/ml)		Mean difference (Δ) in log10 plasma HIV RNA		Mean difference (Δ) in CD4+ T cell counts	
Grade	N	Crude PR (95% CI)	*P*	Crude mean Δ (95% CI)	*P*	Crude mean Δ (95% CI)	*P*
Asymptomatic	262	1.0 (reference)		0.00 (reference)		0.00 (reference)	
SR GH ^†^	101	1.07 (0.92, 1.25)	0.388	+0.10 (−0.05, 0.25)	0.201	−46 (−108, 17)	0.150
1 L-V^‡^	125	1.21 (1.06, 1.38)	0.005	+0.19 (0.05, 0.32)	0.006	−95 (−152, − 37)	0.001
2 L-V	92	1.25 (1.09, 1.44)	0.002	+0.28 (0.12, 0.44)	0.001	−176 (−235, −116)	<0.001
3 L-V	70	1.50 (1.34, 1.68)	<0.001	+0.42 (0.25, 0.58)	<0.001	−273 (−336, −212)	<0.001

Note: GEE, Generalized Estimating Equations; CI, confidence interval; PR, prevalence ratio; *P*, p-value; SR GH, self reported genital herpes; L-V, lesion-visit; ^†^positive self report of genital herpes only; ^‡^number of visits with lesions.

**Table 4:** Adjusted* prevalence risk ratios (PRs) of detectable plasma HIV RNA and mean differences in plasma HIV RNA and CD4+ T cell counts (from the GEE analysis with robust variance estimation) by degree of HSV-2 clinical activity.

		Detectable plasma HIV RNA (>4,000 copies/ml)		Mean difference (Δ) in log10 plasma HIV RNA		Mean difference (Δ) in CD4+ T cell counts	
Grade	N	Adjusted PR (95% CI)	*P*	Adjusted mean Δ (95% CI)	*P*	Adjusted mean Δ (95% CI)	*P*
Asymptomatic	262	1.0 (reference)		0.00 (reference)		0.00 (reference)	
SR GH^†^	101	1.05 (0.90, 1.22)	0.509	+0.11 (−0.03, 0.25)	0.130	−5 (−23, 13)	0.595
1 L-V^‡^	125	1.11 (0.98, 1.27)	0.105	+0.15 (0.02, 0.27)	0.026	−19 (−39, −30)	0.047
2 L-V	92	1.13 (0.98, 1.30)	0.083	+0.16 (0.01, 0.31)	0.034	−32 (−51, −13)	0.001
3 L-V	70	1.21 (1.06, 1.39)	0.005	+0.29 (0.13, 0.44)	<0.001	−51 (−81, −21)	0.001
		Trend *P* = 0.005		Trend *P*<0.001		Trend *P*<0.001	

Note: GEE, Generalized Estimating Equations; CI, confidence interval; PR, prevalence ratio; *P*, p-value; SR GH, self reported genital herpes; L-V, lesion-visit; ^*^adjusted for baseline CD4+ T cell count, herpes medications and type of antiretroviral therapy used at the visit, age, race, lifetime number of sexual partners, injection and noninjection drug use; ^†^positive self report of genital herpes only; ^‡^number of visits with lesions.

**Table 5:** Adjusted* prevalence risk ratios (PRs) of detectable plasma HIV RNA and mean differences in plasma HIV RNA and CD4+ T cell counts (from the cross-sectional log binomial and linear regression models at the 3^rd^ visit) by degree of HSV-2 clinical activity.

		Detectable plasma HIV RNA (>4,000 copies/ml)		Mean difference (Δ) in log10 plasma HIV RNA		Mean difference (Δ) in CD4+ T cell counts	
Grade	N	Adjusted PR (95% CI)	*P*	Adjusted mean Δ (95% CI)	*P*	Adjusted mean Δ (95% CI)	*P*
Asymptomatic	262	1.0 (reference)		0.00 (reference)		0.00 (reference)	
SR GH^†^	101	1.08 (0.89, 1.30)	0.449	+0.18 (−0.006, 0.37)	0.057	−13 (−50, 23)	0.471
1 L-V^‡^	125	1.11 (0.94, 1.32)	0.212	+0.19 (0.02, 0.37)	0.028	−42 (−76, −8)	0.015
2 L-V	92	1.14 (0.96, 1.37)	0.139	+0.21 (0.02, 0.41)	0.029	−64 (−103, −26)	0.001
3 L-V	70	1.32 (1.12, 1.56)	0.001	+0.27 (0.07, 0.46)	0.007	−101 (−144, −59)	<0.001
		Trend *P* = 0.004		Trend *P* = 0.004		Trend *P* = <0.001	

Note: CI, confidence interval; PR, prevalence ratio; *P*, p-value; SR GH, self reported genital herpes; L-V, lesion-visit; ^*^adjusted for baseline CD4+ T cell count, herpes medications and type of antiretroviral therapy used at the visit, age, race, lifetime number of sexual partners, injection and noninjection drug use; ^†^positive self report of genital herpes only; ^‡^number of visits with lesions.

### e) Association between frequency of lesion-visits and mean differences in the levels of detectable plasma HIV RNA and CD4+ T cell levels

In the crude GEE models women with lesions at all three visits were observed to have up to 0.42 (Table 3) and in adjusted models up to 0.29 log_10_ copies/ml higher mean plasma HIV RNA levels compared to women in the asymptomatic group who had no lesion-visits (Table 4, trend p-value <0.001). The average difference in CD4+ T cell counts was up to 273 cells less among women with lesions at all three visits in the crude models compared to women in asymptomatic group (Table 3) but only up to 51 cells less in the multivariate models adjusted for baseline CD4+ T cell count (Table 4, trend p-value <0.001). This difference was up to 101 cells less when linear regression was carried out at the 3^rd^ visit (Table 5, trend p-value <0.001).

## Discussion

Our findings indicate that the levels of HIV replication and immunocompromise are associated with the presence of active HSV-2 infection. Specifically, two sides of the observed association deserve further evaluation: 1) HIV induced CD4+ T cell loss directly correlated with the degree of HSV-2 activity or frequent symptomatic HSV-2 reactivations; and 2) active HSV-2 infection with frequent HSV-2 reactivations was, in turn, associated with modestly elevated plasma HIV RNA levels and reduced CD4+ T cell count over time, compared to women remaining clinically asymptomatic after adjusting for baseline CD4+ T cell levels.

Immunological determinants of HSV-2 infection severity are not completely understood and there are few data on the associations between innate/acquired immune responses and frequencies of HSV-2 reactivation. Moreover, data regarding the relative importance of CD8+ T cells vs. CD4+ T cells as pure effector cells in controlling HSV-2 infection remains controversial [17]–[21]. The correlation of CD4+ T cell depletion with frequency/severity of symptomatic recurrences observed in this study suggests that CD4+ T cells are perhaps one of the important immunologic correlates of protection against HSV-2 reactivations.

While it appears that T cell mediated immunity is of key importance in keeping HSV-2 in check, infiltration and persistence of the same T cells as well as other inflammatory cells at the site of inflammation may in fact provide a perfect milieu for HIV to infect additional CD4+ T cells and thus, enhance HIV replication. Recent *in situ* study of the cellular infiltrates of HSV-2 lesions demonstrated that CD4+ T cells accumulated at the site of lesions had enriched CCR5 expression and were in close proximity to dendritic cells (DCs) expressing DC-SIGN or CD123 of DCs, the receptor known for the ability to transfer HIV to CD4+ T cells [22]. This may present a risk for enhanced local HIV replication and the potential for its systemic dissemination, which perhaps depends on the extent and duration of local inflammation elicited by host immune responses. Indeed, a modest but consistent trend towards elevated plasma HIV RNA levels in symptomatic women compared to asymptomatic women was observed even after adjusting for baseline CD4+ T cell levels. However, it is possible that these changes may have been due to incomplete adjustment of immunosuppression by CD4+ T cells. Despite that, the observed difference of 0.29 log10 copies/ml in plasma HIV RNA load in women with frequent lesion-visits compared to women who had none is very similar to the 0.25 log10 reduction in mean plasma HIV RNA observed in the recent acyclovir suppressive therapy trial [23] which translated into a 16% reduction in HIV disease progression [24]. The fact that the acyclovir experimental trial and our observational study in which most participants did not use acyclovir or other herpes medications found similar magnitude changes in HIV viral load, leads us to speculate whether the frequency and severity of HSV-2 reactivations were responsible for the magnitude of the observed effect in the trial. It is unlikely that the small to modest changes in HIV disease markers associated with HSV-2 reactivations observed in this population will negatively affect the development of AIDS or overall mortality, particularly in the HAART era. On the other hand, these seemingly small viral load changes and reductions in disease progression may not be innocuous. A recent systematic review and meta- analysis of small viral load changes in treatment naïve HIV positive adults showed that even small increases in plasma HIV RNA levels can eventually lead to faster disease progression and a higher risk of ongoing HIV transmission [25]. Although it is possible that the reductions in HIV RNA load observed in clinical trials may have been mediated through the recently discovered inhibitory effect of acyclovir on HIV reverse transcriptase, this effect was noted to be dependent on the ability of HSV-2 and other herpesviruses to provide sufficient amounts of activated acyclovir [26]. This would likely only be achieved if there is sufficient replication of HSV-2 and of other herpesviruses present in the host at the time. Multiple other trials observed similar or greater magnitude reductions in plasma HIV RNA levels with suppressive herpes therapy [27]–[30].

The magnitude of CD4+ T cells lost due to frequent symptomatic HSV-2 reactivations after adjustment for baseline CD4+ T cell levels was not high and may seem to be negligible. However, it is possible that the CD4+ T cell values at the lesion-visits may in fact represent the “elevated” values due to increased activation of CD4+ and other immune cells in response to HSV-2 reactivation which may exacerbate CD4+ T cell loss. In support of this possibility is the observation of higher CD4+ T cell counts among treatment naïve HIV-1/HSV-2 co-infected adults in early HIV infection [31] which may reflect vigorous immune response to HSV-2 reactivations in a not yet deeply immunosuppressed host. Indeed, the trend of CD4+ T cell loss was slight but persisted across the visits and by the time of the last visit those with lesions at all three visits had the lowest mean CD4+ T cell counts compared to those who had none.

A main limitation of the study is the possible misclassification bias due to use of self reported lesions as one of the indicators for the presence of active HSV-2 infection. The WIHS women have not been specifically educated for HSV-2 which can considerably increase the recognition of mild or atypical symptoms [32], [33]. This would be particularly relevant to the asymptomatic group as subsequent follow up data revealed that nearly 75% (n = 196) of 262 asymptomatic women experienced symptoms suggestive of HSV-2 reactivation during extended follow up. Thus, the results of this study are likely to be underestimated as the study did not use HSV-2 uninfected women as controls in whom the possibility of clinical and subclinical reactivations should be minimal if not absent. Another limitation is the absence of laboratory confirmation of genital HSV-2 reactivations in these women. However, laboratory confirmation of HSV-2 reactivations alone is not optimal as HSV-2 detection is influenced by multiple factors such as the intermittent nature of HSV-2 shedding, shedding from multiple anatomic locations as well as the laboratory methodology used. Other limitations include the use of semiannual data that precluded direct assessment of recurrences between the visits and possibly limited generalizability to those with incident HIV and HSV-2 infections where viral and host immune response dynamics are different from those with established infections.

In summary, HIV induced CD4+ T cell loss was associated with symptomatic and frequent HSV-2 reactivations. Symptomatic HSV-2 infection with frequent reactivations, in turn, was associated with modestly elevated HIV replication and reduced CD4+ T cell count over time. Long term studies will be necessary to clarify the exact role the frequency and severity of HSV-2 reactivations play in the natural history of HIV infection, particularly in developing countries where there is no universal access to HAART therapy. In addition, since HSV-2 is most active immediately after HSV-2 acquisition it may be worth to investigate whether early herpes suppressive therapy will delay the need for initiation of HAART in both developing and developed country settings among individuals with frequent HSV-2 reactivations.
